# Physical health challenges faced by elders with severe mental illness: population-based retrospective cohort study

**DOI:** 10.1192/bjo.2024.765

**Published:** 2024-10-15

**Authors:** Chin-Kuo Chang, Richard D. Hayes, Matthew Broadbent, Hitesh Shetty, Yu-Ping Su, Paul D. Meesters, Robert Stewart

**Affiliations:** Global Health Program, College of Public Health, National Taiwan University, Taipei City, Taiwan; Institute of Epidemiology and Preventive Medicine, College of Public Health, National Taiwan University, Taipei City, Taiwan; and Department of Psychological Medicine, King's College London, London, UK; South London and Maudsley NHS Foundation Trust, London, UK; South London and Maudsley NHS Foundation Trust, London, UK; and Department of Psychological Medicine, King's College London, London, UK; South London and Maudsley NHS Foundation Trust, London, UK; Department of Psychiatry, Cathay General Hospital, Taipei City, Taiwan; Department of Research and Education, Friesland Mental Health Services, Leeuwarden, The Netherlands

**Keywords:** Severe mental illness, hospital admission, schizophrenia, bipolar disorder, schizoaffective disorder

## Abstract

**Background:**

Severe mental illness (SMI), which includes schizophrenia, schizoaffective disorder and bipolar disorder, has profound health impacts, even in the elderly.

**Aims:**

To evaluate relative risk of hospital admission and length of hospital stay for physical illness in elders with SMI.

**Method:**

To construct a population-based retrospective cohort observed from April 2007 to March 2016, data from a case registry with full but de-identified electronic health records were retrieved for patients of the South London and Maudsley NHS Foundation Trust, the single secondary mental healthcare service provider in south-east London. We compared participants with SMI aged >60 years old with the general population of the same age and residing in the same areas through data linkage by age-, sex- and fiscal-year-standardised admission ratios (SARs) for primary diagnoses at hospital discharge. Furthermore, we compared the duration of hospital stay with an age-, sex- and cause-of-admission-matched random group by linear regression for major causes of admission.

**Results:**

In total, records for 4175 older people with SMI were obtained, relating to 10 342 admission episodes, showing an overall SAR for all physical illnesses of 5.15 (95% CI: 5.05, 5.25). Among the top causes of admission, SARs ranged from 3.87 for circulatory system disorders (ICD-10 codes: I00–I99) to 6.99 for genitourinary system or urinary conditions (N00–N39). Specifically, the diagnostic group of ‘symptoms, signs and findings, not elsewhere classified’ (R00–R99) had an elevated SAR of 6.56 (95% CI: 6.22, 6.90). Elders with SMI also had significantly longer hospital stays than their counterparts in the general population, especially for digestive system illnesses (K00–K93), after adjusting for confounding.

**Conclusions:**

Poorer overall physical health and specific patterns were identified in elders with SMI.

Severe mental illness (SMI), a group of psychiatric diagnoses including schizophrenia, schizoaffective disorder, and bipolar disorder, is a major public health concern with a substantial impact on many aspects of patients’ daily lives,^1–3^ as well as on medical service provision and family caregivers.^4–6^ The direct and indirect long-term health consequences of SMI are reflected in its profound influence on general health, for which all-cause mortality is commonly used as a comprehensive indicator.^2,6–9^ Studies from various clinical settings and countries, including participants with a wide variety of socioeconomic backgrounds, have reported consistent results of significantly elevated all-cause mortality, with two- to three-fold increases in relative risk leading to a reduction in life expectancy of almost 15 years.^2,3,6–8,10–15^ In past decades, the gap in all-cause mortality between people living with SMI and the general population has persisted or even broadened.^16^ Potential reasons include adverse lifestyle factors, poorer healthcare utilisation, disparities in access to health services and side-effects of long-term psychotropic medication.^17–23^ Among people with SMI, unnatural causes of death (i.e. homicide and suicide) have been found to be of particular importance, especially for those who died at a younger age.^8,14^ However, natural causes of death have been estimated to contribute to as much as 80% of lost life expectancy among people with SMI in comparison with the general population, especially disorders of the circulatory system (mostly cardiovascular diseases), which are the leading cause of death for both sexes.^3,14,15,24^ Even in older individuals, SMI remains associated with two- or three-fold elevated all-cause mortality.^25–27^

Clinical management strategies for the SMI population emphasise the need to deal with increasing mental and physical healthcare requirements.^28,29^ To address this issue, which is of particular research interest, further investigations into healthcare utilisation and disparities in access to health services for people with SMI have been suggested.^4,17,18,30,31^ As well as the psychopathologic manifestations of SMI, research has focused on health challenges met by older people living with SMI, with the ageing of SMI population.^29^ Compared with studies of mortality,^9,26^ studies of physical comorbidities in older people with SMI have had relative small sample sizes; this has meant that only a certain number of major disorders could be analysed because of the limited statistical power,^2,3,7,32–34^ preventing a comprehensive and detailed clinical picture from being obtained. Moreover, inconsistent findings have been reported regarding the risk of hospital admission for physical illness and length of hospital stay, which is a surrogate indicator of clinical severity and complexity at admission.^3,32^ Consequently, the issue of comorbidity in older people with SMI has not been fully addressed.

In this study, we aimed to systematically evaluate the risks of hospital admission across the full range of discharge diagnoses in a cohort consisting of older people with SMI from a population-based psychiatric case registry database in a geographic area of south London, UK. Lengths of hospital stay for major causes of hospital admission were also compared between this cohort and their counterparts in the general population as an indicator of disease severity and clinical complexity at admission.

## Method

### Setting of data source

Data were obtained for four south London boroughs (Croydon, Lambeth, Lewisham and Southwark), the geographically defined catchment area for the South London and Maudsley NHS Foundation Trust (SLaM), which has 1.36 million residents according to the 2011 UK Census. SLaM offers comprehensive secondary mental health services to this catchment area, providing in-patient care, community services, forensic services and liaison services to local general hospitals. Since 2006, all SLaM services have been using electronic mental health records. The SLaM Biomedical Research Centre Case Register was set up as a data resource to enable research use of complete but de-identified data from electronic health records via the Clinical Record Interactive Search (CRIS) platform, launched in 2008.^35^

The authors assert that all procedures contributing to this work comply with the ethical standards of the relevant national and institutional committees on human experimentation and with the Helsinki Declaration of 1975, as revised in 2008. All procedures involving human subjects were approved by the Oxford Research Ethics Committee C (reference number: 23/SC/0257) for secondary data analyses under a rigorous monitoring framework and security model with patient-led governance regulations.

### Study cohort and covariates

Applying a retrospective cohort study design, we constructed a cohort of older patients (>60 years old) with a previous primary or secondary diagnosis of a disorder classified by the World Health Organization ICD-10 under code F20 (schizophrenia), F25 (schizoaffective disorders) or F31 (bipolar disorders) before or during the observation period of 1 Apr 2007 to 31 Mar 2016. Diagnoses were ascertained from structured fields supplemented by a natural language processing algorithm for diagnostic statements in open-text fields developed by the Generalised Architecture for Text Engineering software.^36^ To compare our research outcomes with those of previous studies,^30,37^ the criterion of >60 years old was used to define older people with SMI. Each eligible participant had to be 60 years old or older at the beginning of the observation period (1 Apr 2007) for those whose earliest SMI diagnosis was confirmed before the observation period; on the first date of SMI diagnosis, if the diagnosis was given later in this period; or turning 60 years old or older during follow-up. Sex and ethnicity were also obtained from structured fields in CRIS. Date of admission to a hospital after SMI diagnosis in the observation period and corresponding diagnoses at discharge were retrieved from a linkage to Hospital Episodes Statistics (HES), a national data resource providing details of all admissions to National Health Service (NHS) hospitals in England. The linkage between CRIS and HES data-sets covering the SLaM catchment areas was performed anonymously by the NHS Health and Social Care Information Centre (now NHS Digital).

### Statistical analysis

As the first step, standardised admission ratios (SARs) were calculated for the cohort of older people with SMI served by SLaM, considering age, sex and fiscal year at risk of admission (1 April to 31 March of two consecutive years) as adjusted confounders, compared with the general population in the SLaM catchment area of the same age range as the ‘standard population’ in indirect standardisation. Age strata were classified by 5-year age bands, i.e. 60–64, 65–69,  … , and 90+ years old. Age and sex structure of the standard population was derived from the 2011 UK Census to generate age- and sex-specific admission rates with the hospital admission data from HES linkage in each fiscal year. For each SAR, the numerator was the ‘observed’ number of admissions experienced by CRIS cohort members, whereas the denominator of the SAR was the ‘expected’ number of hospital admission events among the SMI subjects obtained by multiplying the observed admission rate of the standard (i.e. general) population by the number of individuals with SMI and summing over fiscal year–age–sex strata, as a random variable. SARs were first calculated for all primary discharge diagnoses in the observation period and then by sex and ICD-10 chapter of physiologic system of disorders, following a standard approach.^38^ Alternatively, SARs were recalculated by eliminating repeated admissions for the same diagnosis code in a fiscal year as a sensitivity analysis to avoid exaggeration by addressing the potential issue of most of the admissions pertaining to a relatively small group of people.

The second comparison evaluated the association of SMI with the duration of hospital stay (in days) for major causes of admission, defined by the primary diagnosis ICD-10 code at discharge for the first observed admission episode in the observation period. On the basis of disease burden to society and the healthcare system, the top five major categories according to admission numbers comprised respiratory system (ICD-10 codes: J00–J99), mixed group of heterogeneous diagnoses (symptoms, signs and findings not elsewhere classified: R00–R99), circulatory system (I00–I99), genitourinary system: urinary conditions (N00–N39) and digestive system (K00–K93); these were taken forward for further analyses, except the category R00–R99. To avoid violating the independent observation assumption (i.e. every observed subject must be independent from others in a random sample), we only included the first discharge diagnosis for admissions across all years. For each SMI case of first hospital admission, up to four non-SMI comparison subjects with the same primary discharge diagnosis of the first three ICD-10 code characters were randomly selected from the general population of the SLaM catchment area, matched by age within a year and sex. Univariable analyses for estimating the effects of SMI among older people and multivariable analyses for further confounding controls were performed on hospital stay duration in days for each of these major causes of admission by linear regression, with matching addressing the study design issue of dependent data by using ‘clusters’ of matching groups specified in the commands for each linear regression. Besides age and sex, the other adjusted confounders were ethnicity (classified as ‘White’, ‘Black’, ‘south Asian’, ‘east Asian’ and ‘others/mixed/unknown’), discharge method (including ‘clinical advice’, ‘clinical consent’, ‘self-discharged’, ‘death’ and ‘others’) and number of physical comorbidities besides the discharge diagnosis group itself, also retrieved from the HES linkage. All the analyses were conducted using Stata statistical software, version 12.1 (StataCorp., College Station, Texas, USA), and statistical significance was set to 0.05 (alpha level) in two-tailed tests.

## Results

### Study subject characteristics

**Fig. 1:**
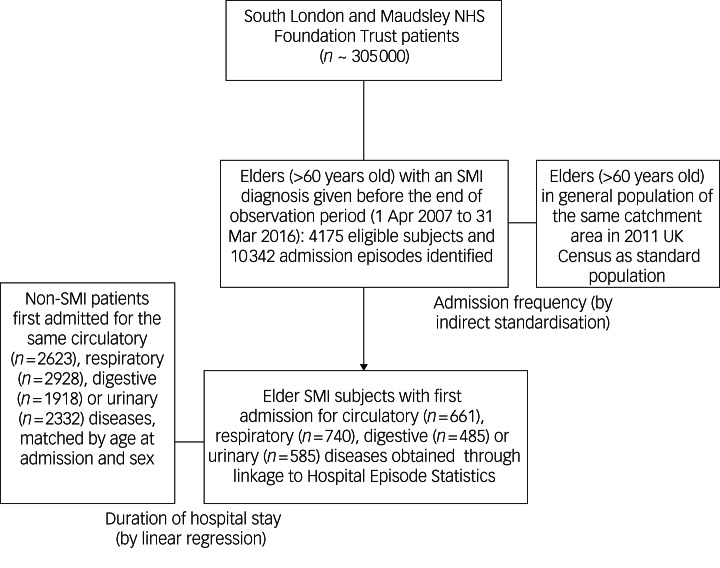
Flowchart of identification of study participants with severe mental illness (SMI) and comparison schemes used in analyses.

Data for a total of 4175 cohort members older than 60 years old and living with SMI during the 9-year observation period with 26 579 person-years contributed were assembled from CRIS, containing electronic health records of over 305 000 SLaM patients at the moment of data retrieval (details shown in Fig. 1). Among them, 1802 were male (43.2%). More details of this study cohort including person-year breakdowns by sex, age group and at-risk fiscal years (1 April 2007 to 31 March 2016) are shown in Supplementary Table 1 available at https://doi.org/10.1192/bjo.2024.765. During the observation period, 10 342 hospital admissions were detected. For all these admission events, 41.6% (4303 admissions) were experienced by males and 58.4% (6039 admissions) by females. For both sexes, the most common cause of first admission was illness of the respiratory system (ICD-10 codes: J00–J99, involving 702 events for males and 954 for females), followed by the group of unspecified conditions: symptoms, signs and findings not elsewhere classified (R00–R99) with 1445 events.

### Relative risks of admission

The overall SAR for our cohort was 5.15 (95% CI: 5.05, 5.25), with 4.82 (95% CI: 4.68, 4.97) for males and 5.41 (95% CI: 5.27, 5.55) for females. SARs ranked according to discharge diagnostic subgroups are displayed in Table 1. As SMI is a group of lifelong conditions, it was not surprising to detect extreme values among the SARs for mental and behaviour disorders (ICD-10 codes: F00–F99), even in the latter life stages. These were excluded from further analyses. Among all physical illnesses, the SAR of 8.49 (95% CI: 7.71, 9.32) for endocrine and metabolic diseases (E00–E90) was particularly notable in terms of effect size (magnitude of relative risk estimated by SAR), followed by urinary conditions (N00–N39), eye conditions (H00–H59) and then respiratory diseases (J00–J99). Among the top five major causes of admissions in terms of the number of admissions (ignoring the group R00–R99), overall SARs ranged from 3.87 for circulatory system (I00–I99) and 4.56 for digestive system (K00–K93) to 6.80 for respiratory system (J00–J99) and 6.99 for genitourinary system: urinary conditions (N00–N39). The SAR for R00–R99 was as high as 6.56 (95% CI: 6.22, 6.90), with 6.02 (95% CI: 5.55, 6.53) for men and 6.99 (95% CI: 6.53, 7.47) for women. In sensitivity analyses excluding multiple admissions for the same discharge diagnosis in a fiscal year (resulting in 15.51% of admissions, *n* = 1604, removed), findings were broadly similar (Table 2, also ranked according to discharge diagnostic subgroup).

**Table 1:** Age-, sex- and fiscal-year-standardised admission ratios by major ICD-10 group for people aged 60 years or older with severe mental illness (1 Apr 2007 to 31 Mar 2016), ranked by number of admissions^a^

Diagnosis in ICD-10	Standardised admission ratio (95% CI^b^; number of admissions)		
	Total (*N* = 4175)	Male (*n* = 1802)	Female (*n* = 2373)
All causes	5.15 (5.05, 5.25; *n* = 10 342)*	4.82 (4.68, 4.97; *n* = 4303)*	5.41 (5.27, 5.55; *n* = 6039)*
Respiratory system (J00–J99)	6.80 (6.47, 7.13; *n* = 1656)*	6.39 (5.92, 6.88; *n* = 702)*	7.14 (6.69, 7.60; *n* = 954)*
Symptoms, signs and findings, not elsewhere classified (R00–R99)	6.56 (6.22, 6.90; *n* = 1445)*	6.02 (5.55, 6.53; *n* = 593)*	6.99 (6.53, 7.47; *n* = 852)*
Circulatory system (I00–I99)	3.87 (3.67, 4.09; *n* = 1312)*	3.54 (3.26, 3.83; *n* = 603)*	4.21 (3.91, 4.53; *n* = 709)*
Genitourinary system: urinary conditions (N00–N39)	6.99 (6.58, 7.43; *n* = 1067)*	5.58 (5.03, 6.16; *n* = 386)*	8.17 (7.57, 8.81; *n* = 681)*
Digestive system (K00–K93)	4.56 (4.26, 4.87; *n* = 881)*	3.78 (3.38, 4.21; *n* = 329)*	5.20 (4.77, 5.65; *n* = 552)*
Injury (S00–T14)	6.34 (5.86, 6.84; *n* = 661)*	6.99 (6.13, 7.94; *n* = 236)*	6.02 (5.46, 6.62; *n* = 425)*
Musculoskeletal system (M00–M99)	2.76 (2.52, 3.02; *n* = 468)*	3.15 (2.70, 3.66; *n* = 173)*	2.58 (2.29, 2.89; *n* = 295)*
Endocrine and metabolic diseases (E00–E90)	8.49 (7.71, 9.32; *n* = 438)*	8.86 (7.64, 10.23; *n* = 186)*	8.24 (7.25, 9.32; *n* = 252)*
Neoplasms (C00-D48)	2.03 (1.84, 2.23; *n* = 425)*	1.83 (1.58, 2.12; *n* = 187)*	2.21 (1.93, 2.51; *n* = 238)*
Mental and behavioural disorders (F00–F99)	20.28 (17.70, 21.60; *n* = 397)*	18.09 (15.57, 20.90; *n* = 184)*	21.08 (18.34, 24.11; *n* = 213)*
Poisoning and other external causes (T15–T98)	5.61 (5.04, 6.22; *n* = 358)*	6.19 (5.31, 7.16; *n* = 179)*	5.13 (4.40, 5.93; *n* = 179)*
Nervous system (G00–G99)	5.83 (5.20, 6.52; *n* = 308)*	5.59 (4.71, 6.58; *n* = 143)*	6.07 (5.17, 7.06; *n* = 165)*
Skin conditions (L00–L99)	6.61 (5.85, 7.45; *n* = 270)*	6.97 (5.81, 8.30; *n* = 127)*	6.32 (5.33, 7.45; *n* = 143)*
Infectious diseases (A00–B99)	5.15 (4.46, 5.91; *n* = 200)*	4.93 (3.92, 6.12; *n* = 82)*	5.31 (4.40, 6.36; *n* = 118)*
Genitourinary system: pelvis, genitals and breasts (N40–N99)	0.87 (0.72, 1.03; *n* = 132)	0.98 (0.76, 1.25; *n* = 68)	0.77 (0.59, 0.98; *n* = 64)*
Blood disorders (D50–D89)	5.36 (4.44, 6.40; *n* = 121)*	5.49 (4.04, 7.31; *n* = 47)*	5.27 (4.14, 6.62; *n* = 74)*
Factors influencing health status and contact with health services (Z00–Z99)	4.64 (3.84, 5.54; *n* = 120)*	4.25 (3.14, 5.62; *n* = 49)*	4.95 (3.86, 6.24; *n* = 71)*
Eye conditions (H00–H59)	6.81 (5.16, 8.82; *n* = 57)*	7.39 (4.73, 10.99; *n* = 24)*	6.44 (4.44, 9.05; *n* = 33)*
Ear conditions (H60–H95)	3.61 (2.23, 5.51; *n* = 21)*	1.81 (0.49, 4.63; *n* = 4)	4.71 (2.74, 7.54; *n* = 17)*
Congenital abnormalities (Q00–Q99)	2.24 (0.73, 5.22; *n* = 5)	1.03 (0.03, 5.73; *n* = 1)	3.17 (0.86, 8.11; *n* = 4)

a. Standard population: residents in London boroughs of Southwark, Croydon, Lambeth and Lewisham according to 2011 UK Census.

b. 95% CI for standardised admission ratio (SAR) = exp[ln(SAR^) ± 1.96 × (1/observed number^1/2^)], where SAR^ is the estimated value of SAR = (observed number of admissions)/(expected number of admissions); *Z*_1−2/α_ = *Z*_0.975_ = 1.96.^55^

* Statistical significance (*P*-value < 0.05).

**Table 2:** Age-, sex- and fiscal-year-standardised admission ratios by major ICD-10 group for people aged 60 years or older with severe mental illness (1 Apr 2007 to 31 Mar 2016), ranked by number of admissions, eliminating repeated admissions in a fiscal year^a^

Diagnosis in ICD-10	Standardised admission ratios (95% CI^b^; number of people with an admission)		
	Total (*N* = 4175)	Male (*n* = 1803)	Female (*n* = 2373)
All causes	5.10 (4.99, 5.21; *n* = 8738)*	4.86 (4.71, 5.02; *n* = 3636)*	5.28 (5.14, 5.43; *n* = 5102)*
Respiratory system (J00–J99)	6.84 (6.47, 7.22; *n* = 1305)*	6.61 (6.07, 7.18; *n* = 558)*	7.01 (6.52, 7.53; *n* = 747)*
Symptoms, signs and findings, not elsewhere classified (R00–R99)	6.37 (6.02, 6.73; *n* = 1233)*	5.87 (5.37, 6.41; *n* = 502)*	6.76 (6.28, 7.27; *n* = 731)*
Circulatory system (I00–I99)	3.82 (3.60, 4.05; *n* = 1090)*	3.44 (3.14, 3.76; *n* = 493)*	4.21 (3.87, 4.56; *n* = 597)*
Genitourinary system: urinary conditions (N00–N39)	7.25 (6.77, 7.76; *n* = 851)*	6.49 (5.81, 7.23; *n* = 329)*	7.83 (7.18, 8.54; *n* = 522)*
Digestive system (K00–K93)	4.39 (4.08, 4.72; *n* = 743)*	3.78 (3.36, 4.25; *n* = 286)*	4.88 (4.44, 5.35; *n* = 457)*
Injury (S00–T14)	6.35 (5.87, 6.87; *n* = 626)*	7.05 (6.16, 8.04; *n* = 223)*	6.02 (5.45, 6.64; *n* = 403)*
Musculoskeletal system (M00–M99)	2.73 (2.48, 2.99; *n* = 438)*	3.07 (2.61, 3.59; *n* = 159)*	2.56 (2.27, 2.88; *n* = 279)*
Endocrine and metabolic diseases (E00–E90)	7.93 (7.14, 8.80; *n* = 361)*	8.34 (7.08, 9.77; *n* = 154)*	7.65 (6.65, 8.77; *n* = 207)*
Neoplasms (C00–D48)	2.17 (1.95, 2.41; *n* = 355)*	2.03 (1.72, 2.37; *n* = 158)*	2.30 (1.99, 2.64; *n* = 197)*
Mental and behavioural disorders (F00–F99)	19.36 (17.37, 21.52; *n* = 343)*	17.30 (14.64, 20.30; *n* = 150)*	21.34 (18.44, 24.57; *n* = 193)*
Poisoning and other external causes (T15–T98)	5.70 (5.08, 6.38; *n* = 306)*	6.11 (5.17, 7.18; *n* = 149)*	5.36 (4.55, 6.27; *n* = 157)*
Nervous system (G00–G99)	5.72 (5.04, 6.45; *n* = 262)*	5.42 (4.49, 6.49; *n* = 118)*	5.98 (5.04, 7.04; *n* = 144)*
Skin conditions (L00–L99)	6.40 (5.60, 7.28; *n* = 231)*	6.76 (5.54, 8.16; *n* = 108)*	6.12 (5.09, 7.30; *n* = 123)*
Infectious diseases (A00–B99)	5.29 (4.55, 6.10; *n* = 186)*	5.14 (4.05, 6.42; *n* = 77)*	5.39 (4.43, 6.51; *n* = 109)*
Genitourinary system: pelvis, genitals and breasts (N40–N99)	1.01 (0.83, 1.20; *n* = 118)	1.20 (0.92, 1.55; *n* = 61)	0.86 (0.65, 1.11; *n* = 57)
Factors influencing health status and contact with health services (Z00–Z99)	4.48 (3.68, 5.40; *n* = 109)*	4.15 (3.03, 5.56; *n* = 45)*	4.74 (3.65, 6.06; *n* = 64)*
Blood disorders (D50–D89)	5.47 (4.48, 6.62; *n* = 106)*	5.26 (3.74, 7.19; *n* = 39)*	5.61 (4.35, 7.12; *n* = 67)*
Eye conditions (H00–H59)	6.74 (5.05, 8.81; *n* = 53)*	7.44 (4.72, 11.16; *n* = 23)*	6.29 (4.24, 8.97; *n* = 30)*
Ear conditions (H60–H95)	3.11 (1.81, 4.99; *n* = 17)*	1.41 (0.29, 4.13; *n* = 3)	4.20 (2.29, 7.04; *n* = 14)*
Congenital abnormalities (Q00–Q99)	2.49 (0.81, 5.80; *n* = 5)	1.15 (0.03, 6.38; *n* = 1)	3.52 (0.96, 9.00; *n* = 4)

a. Standard population: residents of London boroughs of Southwark, Croydon, Lambeth and Lewisham according to 2011 UK Census.

b. 95% CI for standardised admission ratio (SAR) = exp[ln(SAR^) ± 1.96 × (1/observed number^1/2^)], where SAR^ is the estimated value of SAR = (observed number of admissions)/(expected number of admissions); *Z*_1−2/α_ = *Z*_0.975_ = 1.96.^55^

* *P* < 0.05.

### Comparisons of length of first hospital stay

In the analyses on length of first hospital stay in days for major discharge diagnoses (excluding admissions for ICD-10 codes R00–R99, as above), the mean length of first hospital stay for elders with SMI admitted for circulatory system diseases was 12.6 (s.d. = 22.2) days, whereas that for matched non-SMI elders was 9.6 (s.d. = 14.7) days. For other major admission causes, the lengths of stay were 10.6 (s.d. = 17.9) days for digestive system diseases, 13.4 (s.d. = 20.0) days for respiratory system diseases and 12.4 (s.d. = 14.7) days for urinary diseases among elders with SMI, all showing statistical significance in comparison with the matched comparison groups. According to the linear regression, older people with SMI admitted to hospitals stayed 3.10 days longer (95% CI: 1.42, 4.78) on average for illnesses of the circulatory system, 2.76 days longer (95% CI: 1.23, 4.30) for illnesses of the respiratory system, 3.11 days longer (95% CI: 1.49, 4.73) for digestive system illnesses and 1.42 days longer (95% CI: 0.05, 2.79) for genitourinary system illnesses: urinary conditions, after controlling for confounders (Table 3).

**Table 3:** Univariable and multivariable linear regression analyses of duration of hospital stay in days for the first admission during the observation period (1 Apr 2007 to 31 Mar 2016) for major diagnoses of physical illness, comparing elders with severe mental illness (SMI) and non-SMI counterparts matched by age, sex and admission cause

Cause of admission (ICD-10 codes)	Crude		Adjusted^a^	
	*B*-value (95% CI)	*P*-value	*B*-value (95% CI)	*P*-value
Circulatory system (I00–I99)	2.95 (1.22, 4.69)*	<0.01	3.10 (1.42, 4.78)*	<0.01
Respiratory system (J00–J99)	2.04 (0.47, 3.61)*	0.01	2.76 (1.23, 4.30)*	<0.01
Digestive system (K00–K93)	2.75 (1.11, 4.40)*	<0.01	3.11 (1.49, 4.73)*	<0.01
Genitourinary system: urinary conditions (N00–N39)	1.13 (−0.27, 2.54)	0.11	1.42 (0.05, 2.79)*	0.04

a. Adjusted for ethnicity, method of discharge and number of other physical comorbidities.

* *P* < 0.05.

## Discussion

### Main findings

In the present work, we investigated the relation between mental and physical illness using data from the largest single secondary mental health service provider in Western Europe to form a cohort of older people with SMI. Major diagnostic groups for admissions included a diverse range of illnesses of the respiratory, circulatory, urinary and digestive systems, as well as a group of diagnoses for ‘symptoms, signs and findings not elsewhere classified’ covered by the R codes in the ICD-10. After controlling for confounding from age, sex and fiscal year by indirect standardisation, the presence of SMI in older people was found to be related to worse physical health, with nearly five-fold elevated risks of overall hospital admission; endocrine and metabolic disorders were the leading cause of admission, followed by urinary conditions, eye conditions and then respiratory diseases. Length of hospital stay, a surrogate indicator of clinical severity and complexity of conditions at admission, was significantly longer for the major admission causes in our cohort, with potential confounders considered. Although the classification of admission causes was relatively broad, it provided a comprehensive picture of the health challenges faced by elders with SMI. Adding to existing evidence in the literature, we found that SMI profoundly affects individuals throughout life, influencing mental and physical health over time and affecting daily living, as well as being associated with additional physical health challenges during ageing.^1,28,34,39^ Although older people with SMI have survived potential causes of death in earlier life stages, physical illnesses still affect these individuals in later life.^3,5,25,30,40,41^

### Comparison with previous studies and impact on clinical practice

Previous studies have mainly concentrated on mortality or morbidity due to cardiovascular diseases in people with SMI, which have been attributed to obesity and metabolic disorders.^7,13–15^ Unhealthy lifestyles, including smoking, alcohol consumption, lack of exercise and poorer psychosocial functions are widely accepted risk factors for vulnerability in people with SMI.^17–22^ Self-neglect has been proposed as another psychosocial functioning issue that results in higher morbidity and need to be admitted for treatments, as well as higher mortality among people with SMI.^42^ The autonomic nervous system (ANS) is part of the peripheral nervous system innervating the organs and systems of major involuntary physiologic processes throughout the body, with involvement in regulation of body temperature, blood pressure, heart rate, digestion functions, and even emotional and/or behavioural responses. Given the diverse functions of the ANS, autonomic dysfunction has been proposed as an explanation for the wide range of conditions observed in people with SMI, based on evidence.^29,43^ ANS dysfunction, also called autonomic dysfunction, has been found to be associated with increased cardiovascular disease risk in individuals with psychiatric disorders, primarily owing to reduced heart rate variability caused not only by the disorders themselves but also by the use of psychotropic medications across various psychiatric disorders, suggesting a fundamental mechanism for elevation of cardiovascular risk.^43^ Furthermore, long-term use of antipsychotics and frequent polypharmacy for psychotropic medication, including antidepressants, lithium and anticonvulsants,^17,19,23^ may increase adverse effects among people with SMI in later life.^5,17,44^ In practice, somatic complaints of people with SMI often prompt extensive evaluations during hospital admissions, but symptoms/signs remain diagnostically non-specific despite advanced testing ruling out major medical disorders. Clinicians in specialisms besides psychiatry may benefit from additional training in communicating with this specific vulnerable population with clinical complexity, as poor psychosocial functioning can make diagnosis difficult. How to minimise impacts of these metabolic effects of psychotropic medication as well as direct/indirect influences of autonomic dysfunction in older people with SMI are challenges for clinicians and caregivers.

### Explanation of outcomes

Concerning eye conditions, visual processing impairments have been well characterised in schizophrenia and discussed for decades, and there is some evidence of common genetic factors.^45^ As well as ocular disorders secondary to diabetes and hypertension, elevated risks of nystagmus, strabismus and poorer visual acuity have been noted among people with schizophrenia.^46^ An increased relative risk of ocular neurovascular conditions, especially glaucoma, was found among people with bipolar disorder, major depressive disorder and schizophrenia in a population-based study in Taiwan,^47^ consistent with our findings.

Well-characterised side-effects of long-term use of antipsychotics are known to increase the risks of respiratory, endocrine, digestive and urinary conditions.^17,20,48^ However, our research also revealed an increased risk of admission for unspecified symptoms or signs (ICD-10 codes: R00–R99), which has not been specifically reported elsewhere in the literature; this warrants further investigations of these non-specific causes of admissions in older people with SMI. As well as the adverse effects of chronic exposure to antipsychotics and unhealthy lifestyle factors, autonomic dysfunction, with concurrent increases in sympathetic activity and decreases in parasympathetic activity caused by SMI itself and psychotropic medications, may further worsen the physical health of people with SMI.^48^ The vagus nerve could be of particular importance with respect to digestive comorbidities in the SMI population, as it is part of the ANS and functions as a major mediator of bidirectional communication in the gut–brain axis, regulating the digestive functions of regional gut motility, digestive juice secretion, duct wall permeability, and even mucosal immune responses, with subtle influences on the composition and activity of the gut microbiome.^49^ In summary, although autonomic dysfunction has been partially implicated in physical illnesses of people with SMI, further investigations into the underlying mechanisms for each significant physical health concern are warranted.

### Advantages and disadvantages

A key strength of our research was the population-based data source with nearly complete coverage of the population in the catchment areas, indicating that this sample of older people with SMI was representative of those in the urban/suburban UK population. Use of natural language processing techniques to ascertain inclusion diagnoses further strengthened this advantage.^36^ The temporal relationship between pre-existing SMI and consequent admission events was adequately established with a retrospective cohort study design. In addition, there was fairly complete ascertainment of hospital admissions covering all secondary NHS healthcare services in England obtained by linking to HES. The other advantage was that we compared people with SMI inclusively with the general population, resulting in more conservative estimation of the relative risk of admission. Regarding limitations, first, the research cohort was constructed based on a case registry and thus used data that were not collected for research purposes. Thus, information might have been incomplete or of uncertain quality, especially with respect to trajectories of treatment and time-varying exposures to psychotropic medication among older cohort members with SMI. Second, the classification of admission causes may have been a slightly crude means of identifying specific admission causes of clinical importance, restricting the possibility of further exploration of those diseases.^3,33^ In addition, the generalisability of our results could be constrained by the sample being limited to an urban and suburban catchment area in south London; the findings need to be more widely replicated by studies in various settings in the future. Moreover, the results of indirect standardisation were not directly comparable across studies, which further restricted the explainability of the current analyses. Last, no confounders other than age, sex and year were controlled in the process of indirect standardisation for SARs, for instance, smoking, drinking and obesity. The potential for overestimation of the SARs should be borne in mind in any further implementation or policy-making. Nonetheless, in the analysis of length of hospital stay in days, we further controlled for ethnicity, method of discharge and number of other physical comorbidities as potential confounders in addition to the matched ones (age, sex and admission cause).

### Public health implications and future directions

The findings of our study underline the importance of monitoring the physical health status of older people with SMI in the community, rather than focusing only on psychiatry treatments.^29^ Early detection, followed by effective intervention to prevent hospital admission, is critical for this vulnerable group. The coverage and frequency of screening projects for illnesses of critical concern in older people should be enhanced, particularly for those with SMI.^50,51^ Improving healthcare services for these individuals requires a well-integrated health management system with organised coordination of health promotion activities, highly accessible primary care, and timely referrals to secondary healthcare services with continuity.^3,30,52,53^ The provision of appropriate primary care and health promotion for older people with SMI will help health service providers to meet their persistent needs with respect to both physical and mental healthcare.^29,34^ Future work should investigate potential barriers to access to appropriate health management programmes, as well as the delivery of timely primary and secondary medical services.^31,52,54^ Research on the role of autonomic dysfunction in SMI treatments and illnesses is warranted as well.^29,43^

## Supporting information

Chang et al. supplementary material Chang et al. supplementary material

## Data Availability

From 3 months to 5 years following the publication of this article, data of individual participants will be available to researchers who provide a methodologically sound research proposal with approval. Data collected for all of the individual participants involved in current analyses will be shared under the UK regulations for anonymised electronic health records. Our detailed study protocol and statistical analysis plan will also be available. Requests and proposals should be directed to chinkuochang@ntu.edu.tw. To gain access, data requestors must sign a data access agreement for legal reasons.
