# Supplementary material for: Physical health challenges faced by elders with severe mental illness: population-based retrospective cohort study
Source: BJPsych Open. 2024 Oct 15;10(6):e178. doi: 10.1192/bjo.2024.765 (PMC11536298; doi:10.1192/bjo.2024.765)
Supplement: Chang et al. supplementary material [file S2056472424007658sup001.docx]

Supplementary Table 1. Demographic characteristics and at-risk fiscal years (1 April 2007 – 31 March 2016) of South London and Maudsley NHS Foundation Trust service users equal or older than 60 years old and with severe mental illness (26,579 person-years observed in total)

| Variable | Contributed person-years | Percentage |
| --- | --- | --- |
| Sex  Female  Male | 15,592  10,987 | 58.66%  41.34% |
| Age group (years old)  60-64  65-69  70-74  75-79  80-84  85-89  90+ | 7,616  6,021  4,943  3,880  2,549  1,117  453 | 28.65%  22.65%  18.60%  14.60%  9.59%  4.20%  1.70% |
| At-risk fiscal year  2007  2008  2009  2010  2011  2012  2013  2014  2015 | 2,682  2,814  2,869  2,929  2,959  3,005  3,050  3,115  3,156 | 10.09%  10.58%  10.79%  10.25%  11.13%  11.31%  11.48%  11.72%  11.87% |
